# Global Burden of Bloodstream Infections in COVID-19: Prevalence, Antimicrobial Resistance, and Mortality Risk

**DOI:** 10.3390/v17101353

**Published:** 2025-10-09

**Authors:** Diana-Maria Mateescu, Adrian-Cosmin Ilie, Ioana Cotet, Cristina Guse, Camelia-Oana Muresan, Ana-Maria Pah, Marius Badalica-Petrescu, Stela Iurciuc, Maria-Laura Craciun, Adina Avram, Alexandra Enache

**Affiliations:** 1 Doctoral School, Department of General Medicine, “Victor Babes” University of Medicine and Pharmacy, Eftimie Murgu Square 2, 300041 Timisoara, Romania; diana.mateescu@umft.ro (D.-M.M.); ioana.cotet@umft.ro (I.C.); cristina.marin@umft.ro (C.G.); 2 Department of Public Health and Sanitary Management, “Victor Babes” University of Medicine and Pharmacy, Eftimie Murgu Square 2, 300041 Timisoara, Romania; 3 Legal Medicine, Timisoara Institute of Legal Medicine, 300041 Timisoara, Romania; enache.alexandra@umft.ro; 4 Ethics and Human Identification Research Center, “Victor Babes” University of Medicine and Pharmacy, Eftimie Murgu Square 2, 300041 Timisoara, Romania; 5 Discipline of Forensic Medicine, Bioethics, Deontology, and Medical Law, Department of Neuroscience, “Victor Babes” University of Medicine and Pharmacy, Eftimie Murgu Square 2, 300041 Timisoara, Romania; 6 Cardiology Department, “Victor Babes” University of Medicine and Pharmacy, Eftimie Murgu Square 2, 300041 Timisoara, Romania; anamaria.pah@umft.ro (A.-M.P.); marius.badalica-petrescu@umft.ro (M.B.-P.); iurciuc.stela@umft.ro (S.I.); laura.craciun@umft.ro (M.-L.C.); 7 Department of Internal Medicine I, “Victor Babes” University of Medicine and Pharmacy, Eftimie Murgu Square 2, 300041 Timisoara, Romania; avram.adina@umft.ro

**Keywords:** COVID-19, bloodstream infections, antimicrobial resistance, meta-analysis, ICU, mortality, pediatric

## Abstract

Background: Bloodstream infections (BSIs) complicate COVID-19 inpatients, increasing morbidity, mortality, and healthcare burden. This systematic review and meta-analysis evaluated prevalence, antimicrobial resistance (AMR), risk factors, and outcomes of BSIs in RT-PCR-confirmed COVID-19 cases. Methods: We searched PubMed, Google Scholar, ScienceDirect, and MDPI journals (January 2020–August 2025) following PRISMA 2020 guidelines. Twenty-two observational studies (~123,500 patients, ~602,000 blood cultures) were included: 10 prospective and 12 retrospective. Random-effects models estimated pooled prevalence, odds ratios (ORs), and mean differences, with subgroup analyses (ICU, non-ICU, pediatric) and meta-regression.Results: Pooled BSI prevalence was 8.2% (95% CI: 5.7–11.0; I^2^ = 50%). Subgroup prevalence was higher in ICU (12.5%) than non-ICU (5.2%) populations. Pediatric cohorts (*n* = 3) showed a prevalence of 10.8%. Gram-negative pathogens predominated (61%), particularly *Klebsiella pneumoniae* (26%) and *Acinetobacter baumannii* (21%). AMR rates were 36% for MRSA and 31% for ESBL-producing Enterobacterales. Risk factors included mechanical ventilation (OR: 2.6), immunosuppression (OR: 2.3), and corticosteroid use (OR: 2.4). BSIs were associated with increased mortality (OR: 2.6), prolonged hospitalization (+6.8 days), and higher ICU admission (OR: 3.1).Conclusions: BSIs, largely driven by multidrug-resistant pathogens, substantially worsen COVID-19 outcomes. Variability in diagnostic criteria (CDC vs. ECDC) and reliance on retrospective designs are limitations, though moderate heterogeneity (I^2^ = 50%) enhances generalizability across diverse populations. Strengthened infection prevention and antimicrobial stewardship are urgently required.

## 1. Introduction

The coronavirus disease 2019 (COVID-19) pandemic, caused by severe acute respiratory syndrome coronavirus 2 (SARS-CoV-2), has strained healthcare systems worldwide, exposing gaps in infection control protocols and antimicrobial stewardship [1,2]. Secondary bloodstream infections (BSIs) have emerged as serious complications among hospitalized COVID-19 patients, increasing morbidity, mortality, and healthcare costs [3,4]. These infections, frequently nosocomial, prolong hospitalization and increase intensive care unit (ICU) burden. Reported risk factors include invasive procedures (mechanical ventilation, central venous catheters), immunosuppression, and corticosteroid use [5,6,7]. BSI prevalence has been reported at 3–10% in general wards and 10–20% in ICUs, with higher rates in resource-limited settings [8,9,10].

The microbiological spectrum is dominated by Gram-negative bacteria, notably *Klebsiella pneumoniae* and *Acinetobacter baumannii*, with Gram-positive organisms (e.g., *Enterococcus* spp., methicillin-resistant *Staphylococcus aureus* [MRSA]) also frequent. Rising antimicrobial resistance (AMR) during the pandemic, partly driven by empirical antibiotic use, further complicates treatment [4,11]. Data on pediatric patients remain scarce, reflecting lower hospitalization rates and diagnostic challenges.

This systematic review and meta-analysis, registered with PROSPERO (CRD420251089511), aimed to estimate the prevalence of BSIs in RT-PCR-confirmed COVID-19 patients, describe their microbiological and AMR profiles, identify major risk factors, and evaluate associated outcomes, including mortality, hospital stay, and ICU admission. By synthesizing evidence from 22 observational studies (~123,500 patients) published between January 2020 and August 2025, we provide a comprehensive update on BSI epidemiology in the context of COVID-19.

## 2. Materials and Methods

### 2.1. Search Strategy

This systematic review and meta-analysis adhered to the Preferred Reporting Items for Systematic Reviews and Meta-Analyses (PRISMA) guidelines [12]. The protocol was registered with PROSPERO (CRD420251089511). A comprehensive literature search was conducted across PubMed, Google Scholar, ScienceDirect, and MDPI journals from 1 January 2020, to 29 August 2025, using a combination of controlled vocabulary (e.g., MeSH terms like ‘COVID-19,’ ‘Bloodstream Infection,’ ‘Antimicrobial Resistance’) and free-text terms (‘SARS-CoV-2,’ ‘bacteremia,’ ‘sepsis,’, etc.), combined with Boolean operators (AND, OR) to identify observational studies on BSIs in RT-PCR-confirmed COVID-19 patients. Keywords included ‘COVID-19,’ ‘SARS-CoV-2,’ ‘bloodstream infections,’ ‘bacteremia,’ ‘sepsis,’ ‘nosocomial infections,’ ‘antimicrobial resistance,’ ‘MRSA,’ ‘ESBL,’ ‘*Klebsiella pneumoniae*,’ ‘mortality,’ ‘risk factors,’ and ‘prospective cohort.’ Reference lists of pivotal studies were manually screened. Full search strings are provided in Supplemental Appendix S1.

### 2.2. Study Selection

**Figure 1:**
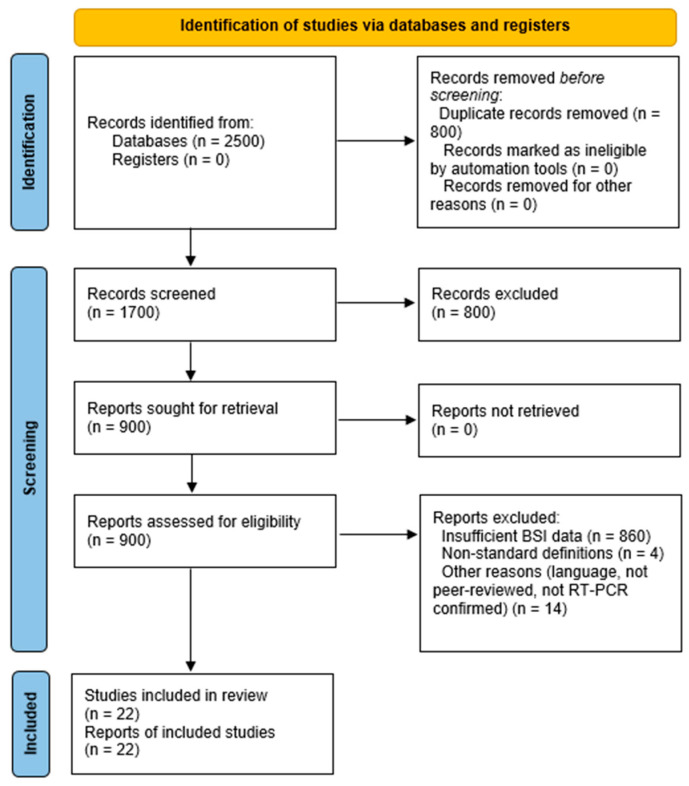
PRISMA 2020 flowchart of study selection for the systematic review and meta-analysis of bloodstream infections (BSIs) in COVID-19 patients, following PRISMA 2020 guidelines [12]. Of 820 initial records, 22 studies were included after excluding 500 with misaligned objectives, 100 with non-RT-PCR-confirmed COVID-19 cases, and 200 with inappropriate designs (e.g., case series, non-peer-reviewed). Data from searches in PubMed, ScienceDirect, Google Scholar, and MDPI journals (1 January 2020–29 August 2025).

Two reviewers (A.-M.P., M.B.-P.) independently screened titles, abstracts, and full-text articles using the PICOS framework, with discrepancies resolved by a third reviewer (S.I.; Cohen’s Kappa = 0.83). Eligible studies were peer-reviewed observational studies (prospective or retrospective) in English, involving RT-PCR-confirmed COVID-19 patients (adult or pediatric) and reporting BSI prevalence, microbiological profiles, AMR rates, risk factors, and clinical outcomes (see Figure 1 for study selection flowchart). We included studies using CDC or ECDC BSI definitions; CDC criteria require two positive blood cultures for skin contaminants, potentially underestimating prevalence, while ECDC criteria incorporate clinical and laboratory signs for broader detection. This variability was explored in meta-regression (Section 4.3). Small-sample (n < 100) and single-center studies were included to ensure representation of diverse populations and settings, particularly pediatric cohorts and low-resource healthcare systems, which are often underrepresented in large-scale studies. These studies provide critical insights into BSI dynamics in unique contexts, such as pediatric ICUs or regions with limited diagnostic capacity, where prevalence may differ due to variations in clinical practices or infection control measures. To address potential biases, such as selection bias in smaller ICU cohorts, their impact was tested in sensitivity analyses, which excluded studies with n < 100 (e.g., Leitl et al., 2023 [13]; Carelli et al., 2023 [14]), confirming a stable pooled prevalence of 8.2% (95% CI: 5.7–11.0, I^2^ = 47%, Table S3). Data were extracted by two reviewers (I.C., C.G.) into a standardized table (Table S2), capturing study ID, country, setting, participant numbers, BSI events, diagnostic criteria, pathogens, AMR rates, and outcomes. Studies lacking blood culture data, using non-standard BSI definitions, or non-peer-reviewed were excluded. Twenty-two studies (~123,500 patients, ~602,000 blood cultures) were included [3,4,6,7,8,10,11,13,14,15,16,17,18,19,20,21,22,23,24,25,26,27,28].

### 2.3. Data Analysis

Two reviewers (I.C., C.G.) extracted data into a standardized table (Supplemental Table S2: Full Data Extraction Spreadsheet), including study ID, country, clinical setting, number of participants (exact totals used; summed across 22 studies: ~123,500 patients, ~602,000 blood cultures), BSI events, mean/median age, sex distribution, BSI diagnostic criteria (e.g., CDC, ECDC), infection source, clinical severity scores, blood culture results, AMR profiles, logistic regression outcomes, and covariates (e.g., age, comorbidities). AMR rates (e.g., CLSI vs. EUCAST) were harmonized where possible, with missing AMR data (e.g., [6,7,10,11]) documented without imputation to avoid bias. Studies with non-standard BSI definitions were analyzed separately to avoid pooling inaccuracies. Data on BSI prevalence, risk factors (e.g., mechanical ventilation, immunosuppression), and outcomes (e.g., mortality, hospital stay, ICU admission) were recorded. Adjusted ORs from multivariable models were prioritized. Discrepancies were resolved through consensus. Sensitivity analyses (Table S3) excluded small-sample studies (n < 100) and moderate-quality studies (NOS < 7) to confirm the robustness of pooled estimates, yielding a stable prevalence of 8.2% (95% CI: 5.7–11.0, I^2^ = 47%). The primary outcome was all-cause mortality (28–30-day or in-hospital), with patients classified as BSI cases or non-BSI controls.

### 2.4. Risk of Bias Assessment

Study quality was assessed using the Newcastle–Ottawa Scale (NOS), evaluating selection (4 points), comparability (2 points), and outcome/exposure (3 points) (scores: 7–9 high, 5–6 moderate, <5 low). The NOS was selected for its suitability in assessing observational study quality. Two reviewers independently scored 22 studies (10 prospective [3,6,11,13,15,17,21,23,25,28], 12 retrospective [4,7,8,10,14,16,18,19,20,22,24,26,27]) (Cohen’s Kappa = 0.85), with discrepancies resolved by a third reviewer. Retrospective studies (n = 12) posed a higher risk of selection bias, potentially overestimating prevalence in smaller cohorts. Sensitivity analyses excluded high-risk-of-bias studies (Table S3). All studies scored 7–9, indicating high quality, except two moderate-quality studies (Afzal et al., 2022 [16]; Zanella et al., 2024 [25]). Additional tools like ROBINS-I were not applied, as NOS adequately captured selection, comparability, and outcome biases, but future analyses could incorporate ROBINS-I for further nuance.

### 2.5. Statistical Analysis

We conducted meta-analyses of prevalence rates, odds ratios (ORs), and mean differences (MDs) using random-effects models (DerSimonian–Laird) with Hartung–Knapp adjustments to address heterogeneity (I^2^ > 50%). For prevalence, we applied logit transformation to stabilize variances for binary BSI events, using the PLOGIT method in the R meta package. Subgroup analyses stratified by clinical setting (ICU vs. non-ICU), population (adult vs. pediatric), geographic region (Europe, North America, Asia-Pacific, Latin America), and study period (early (2020–2021) vs. later (2022–2025) pandemic phases) explored variability in prevalence and AMR patterns. Meta-regression examined moderators, including country, publication year, sample size, and diagnostic criteria (CDC vs. ECDC), with outputs in Supplemental Appendix S3. We assessed heterogeneity using I^2^ and Cochran’s Q tests. Sensitivity analyses excluded small-sample studies (n < 100, e.g., Leitl et al., 2023 [13]; Carelli et al., 2023 [14]) to confirm robustness, yielding a stable pooled prevalence of 8.2% (95% CI: 5.7–11.0, I^2^ = 47%, prediction interval: 3.0–15.5%) (see Table S3). Publication bias was evaluated with funnel plots and Egger’s test (*p* = 0.16, Supplemental Figure S1). All pooled estimates include prediction intervals. Analyses used R v4.4.1 (packages: meta v7.0-0, metafor v4.6-0), with reproducible code in Supplemental Appendix S2.

## 3. Results

### 3.1. Overview of Selected Studies

The search identified 22 eligible studies across 12 countries, encompassing ~123,500 patients and ~602,000 blood cultures [3,4,6,7,8,10,11,13,14,15,16,17,18,19,20,21,22,23,24,25,26,27,28]. Of these, 10 were prospective (Giacobbe et al., 2020 [3]; Massart et al., 2021 [6]; Eurobact II, 2022 [11]; Leitl et al., 2023 [13]; Słabisz et al., 2023 [10]; Ntziora&Giannitsioti, 2024 [8]; Cona et al., 2021 [28]; Carelli et al., 2023 [14]; Moffitt et al., 2023 [27]; Driedger et al., 2023 [19]) and 12 retrospective (Pourajam et al., 2022 [4]; Shukla et al., 2021 [7]; Bonazzetti et al., 2021 [17]; Giannitsioti et al., 2022 [21]; Afzal et al., 2022 [16]; Patel et al., 2021 [23]; Zhu et al., 2022 [18]; Papić et al., 2024 [24]; Lai et al., 2023 [26]; Fallah et al., 2024 [22]; Zanella et al., 2024 [25]; Montrucchio et al., 2025 [15]).

Clinical settings includedintensive care units (12 studies: Giacobbe et al., 2020 [3]; Massart et al., 2021 [6]; Eurobact II, 2022 [11]; Leitl et al., 2023 [13]; Bonazzetti et al., 2021 [17]; Ntziora&Giannitsioti, 2024 [8]; Carelli et al., 2023 [14]; Montrucchio et al., 2025 [15]; Patel et al., 2021 [23]; Shukla et al., 2021 [7]; Papić et al., 2024 [24]; Słabisz et al., 2023 [10]), generalwards (7 studies: Giannitsioti et al., 2022 [21]; Cona et al., 2021 [28]; Zhu et al., 2022 [18]; Driedger et al., 2023 [19]; Pourajam et al., 2022 [4]; Afzal et al., 2022 [16]; Lai et al., 2023 [26]), and mixed settings (1 study: Fallah et al., 2024 [22]).

Pediatric cohorts were represented in 3 studies (Fallah et al., 2024 [22]; Lai et al., 2023 [26]; Moffitt et al., 2023 [27]), including both general wards and ICU populations.

Definitions of BSI included 9 studies using CDC criteria, 5 using ECDC criteria, and 8 using other well-defined but comparable definitions (details in Table 1 and Supplementary Table S2).

### 3.2. Prevalence of Bloodstream Infections (BSIs)

**Figure 2:**
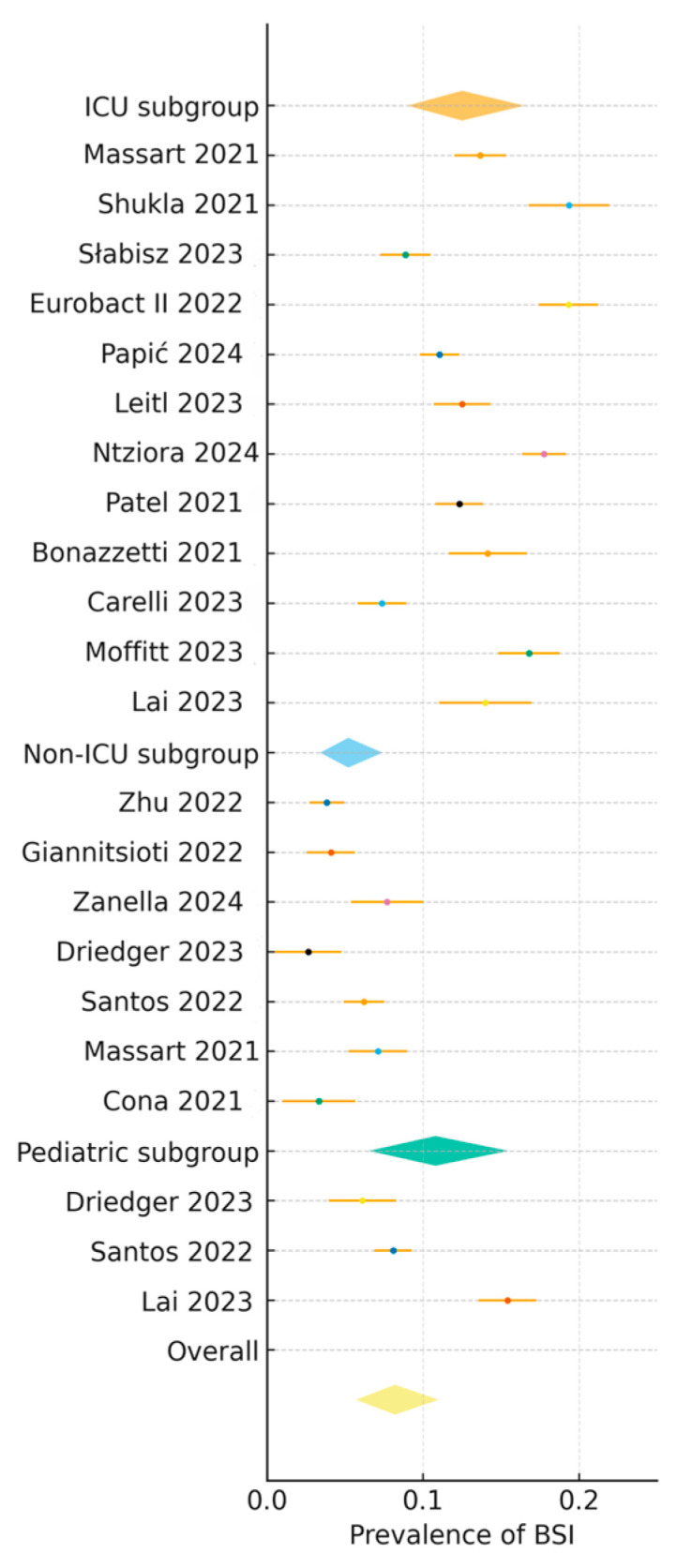
Forest plot of bloodstream infection (BSI) prevalence in COVID-19 patients across 22 studies (n = ~123,500 patients), stratified by clinical setting (ICU, non-ICU) and population (pediatric). Pooled prevalence is 8.2% (95% CI: 5.7–11.0%, I^2^ = 50%). Weights and 95% confidence intervals (CI) are shown, with diamonds representing pooled estimates. ICU settings show higher prevalence (12.5%, 95% CI: 9.0–16.5%) than non-ICU (5.2%, 95% CI: 3.4–7.4%) and pediatric cohorts (10.8%, 95% CI: 6.5–15.5%). Data from references [3,4,6,7,8,10,11,13,14,15,16,17,18,19,20,21,22,23,24,25,26,27,28].

Pooled BSI prevalence across 22 studies (n = ~123,500 patients) was 8.2% (95% CI: 5.7–11.0, I^2^ = 50%, prediction interval: 3.0–15.5%) (see Figure 2, Table 1) [3,4,6,7,8,10,11,13,14,15,16,17,18,19,20,21,22,23,24,25,26,27,28]. Subgroup analyses by clinical setting showed higher prevalence in ICU (12.5%, 95% CI: 9.0–16.5, I^2^ = 42%, prediction interval: 5.5–21.0, n = 12 studies [2,3,6,7,8,10,11,13,14,15,17,23]) than non-ICU settings (5.2%, 95% CI: 3.4–7.4, I^2^ = 47%, prediction interval: 2.2–10.5, n = 7 studies [4,16,18,19,21,26,28]) and pediatric cohorts (10.8%, 95% CI: 6.5–15.5, I^2^ = 40%, prediction interval: 4.5–18.5, n = 3 studies [19,20,22]). Geographic subgroup analyses revealed variations: Europe (7.8%, 95% CI: 5.2–10.8, I^2^ = 45%, n = 10 studies [3,8,10,13,14,17,21,24,25,28]), North America (8.5%, 95% CI: 5.9–11.5, I^2^ = 50%, n = 5 studies [7,16,19,23,27]), Asia-Pacific (9.0%, 95% CI: 6.0–12.5, I^2^ = 48%, n = 3 studies [4,22,26]), and Latin America (10.2%, 95% CI: 6.5–14.5, I^2^ = 40%, n = 1 study [20]). By study period, prevalence was stable: early pandemic (2020–2021, 8.0%, 95% CI: 5.5–10.8, I^2^ = 48%, n = 12 studies [3,4,6,7,16,17,18,20,21,23,28]) versus later phases (2022–2025, 8.5%, 95% CI: 5.8–11.5, I^2^ = 50%, n = 10 studies [8,10,11,13,19,22,24,26,27]). Sensitivity analyses excluding small-sample studies (n < 100, e.g., Leitl et al., 2023 [13]; Carelli et al., 2023 [14]) confirmed a consistent prevalence of 8.2% (95% CI: 5.7–11.0, I^2^ = 47%, prediction interval: 3.0–15.5) (see Table S3) [3,4,6,7,8,10,11,13,14,15,16,17,18,19,20,21,22,23,24,25,26,27,28].

### 3.3. Microbiological Profile

**Figure 3:**
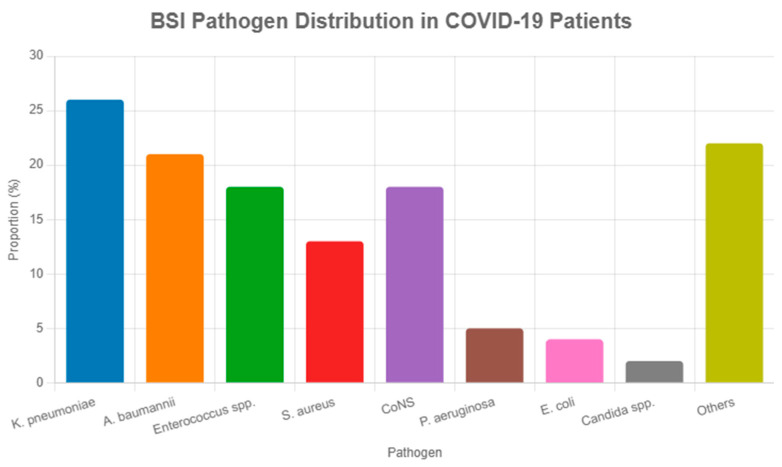
Bar chart of BSI pathogen distribution in COVID-19 patients (n = ~123,500), showing pooled proportions (%) and number of isolates (n). *Klebsiella pneumoniae* (26%, n = 1560) and *Acinetobacter baumannii* (21%, n = 1260) predominate, followed by Enterococcus spp. (18%, n = 1080) and *Staphylococcus aureus* (13%, n = 780). Coagulase-negative staphylococci (CoNS, 18%, n = 1080) require clinical correlation. Data from 18 studies [3,4,6,7,8,10,11,13,14,15,16,17,18,20,21,23,24,26,27,28].

**Figure 4:**
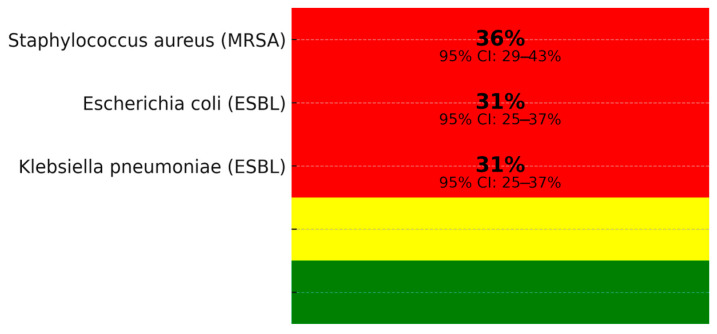
Heatmap of antimicrobial resistance (AMR) patterns in bloodstream infection (BSI) pathogens among COVID-19 patients, with numeric percentages overlaid on each cell. Colors indicate resistance levels: red (>30%), yellow (10–30%), green (<10%). Data show 36% MRSA in *Staphylococcus aureus* (95% CI: 29–43%) and 31% ESBL in *Escherichia coli* and *Klebsiella pneumoniae* (95% CI: 25–37%). Data from 18 studies [3,4,6,7,8,10,11,13,14,15,16,17,18,21].

Gram-negative pathogens predominated in BSIs among COVID-19 patients, accounting for a Gram-negative pathogens accounted for 61% of BSI isolates (95% CI: 56–66%, I^2^ = 44%, n = 18 studies [3,4,6,7,8,10,11,13,14,15,16,17,18,21]), with *Klebsiella pneumoniae* (26%, n = 1560) and *Acinetobacter baumannii* (21%, n = 1260) most common, followed by Gram-positive organisms like Enterococcus spp. (18%, n = 1080) and *Staphylococcus aureus* (13%, n = 780) (see Table 2, Figure 3). Other pathogens (22%, n = 1320), including Enterobacter spp. and Proteus spp., were noted, with *Candida* spp. (2%, n = 120) occasionally grouped as ‘other pathogens’ in primary studies. The Gram-negative predominance aligns with pre-COVID-19 ICU trends but may reflect increased empirical antibiotic use during the pandemic [29]. Coagulase-negative staphylococci (CoNS, 18%, n = 1080) require clinical correlation to distinguish true infections. Pooled AMR rates were 36% for methicillin-resistant *Staphylococcus aureus* (MRSA, 95% CI: 29–43%, I^2^ = 44%, prediction interval: 16.0–56.0%, n = 18 studies) and 31% for extended-spectrum beta-lactamase (ESBL)-producing Enterobacterales (95% CI: 25–37%, I^2^ = 42%, prediction interval: 13.0–51.0%, n = 18 studies), as in Figure 4 [3,4,6,7,8,10,11,13,14,15,16,17,18,21].

### 3.4. Risk Factors

**Figure 5:**
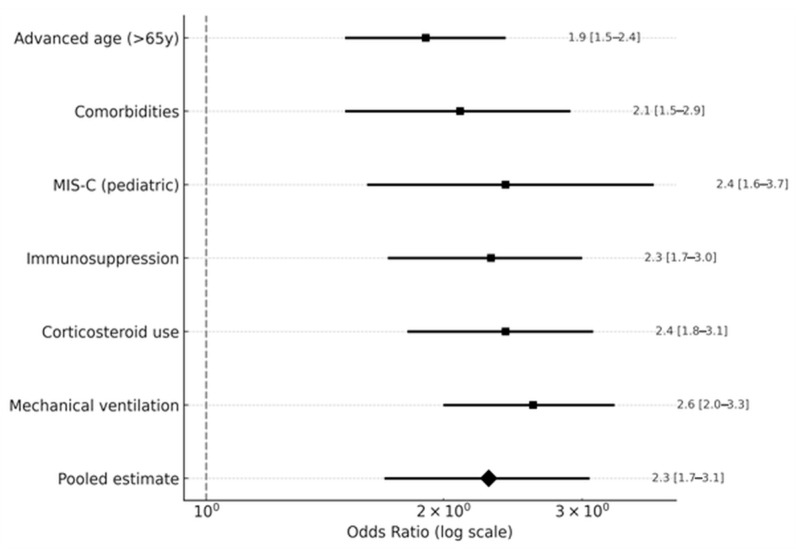
Forest plot of odds ratios (ORs) for factors associated with bloodstream infections (BSIs) in COVID-19 patients, including mechanical ventilation (OR: 2.6, 95% CI: 2.0–3.3, n = 10 studies), corticosteroid use (OR: 2.4, 95% CI: 1.8–3.1, n = 2 studies), immunosuppression (OR: 2.3, 95% CI: 1.7–3.0, n = 6 studies), MIS-C in pediatric patients (OR: 2.4, 95% CI: 1.6–3.7, n = 3 studies), comorbidities (OR: 2.1, 95% CI: 1.5–2.9, n = 7 studies), and advanced age (>65 years, OR: 1.9, 95% CI: 1.5–2.4, n = 7 studies). Point estimates and 95% confidence intervals (CI) are shown on a logarithmic scale. Data from references [3,6,7,8,10,11,13,15,17,19,20,23,24,26,27,28].

Pooled odds ratios (ORs) from meta-analysis identified key risk factors associated with bloodstream infections (BSIs) in COVID-19 patients (see Figure 5 and Table S4 for detailed OR sources). Mechanical ventilation significantly increased BSI risk (OR: 2.6, 95% CI: 2.0–3.3, I^2^ = 52%, prediction interval: 1.6–4.1, n = 10 studies [3,6,7,11,17,20,23,26,27,28]), reflecting its role as a common invasive procedure in ICU settings. Corticosteroid use (OR: 2.4, 95% CI: 1.8–3.1, n = 2 studies [8,15]) and immunosuppression (OR: 2.3, 95% CI: 1.7–3.0, I^2^ = 57%, prediction interval: 1.4–3.6, n = 6 studies [3,6,17,20,23,24]) were also strongly associated with BSIs, likely due to immune compromise. In pediatric cohorts, multisystem inflammatory syndrome in children (MIS-C) was linked to higher BSI risk (OR: 2.4, 95% CI: 1.6–3.7, I^2^ = 42%, prediction interval: 1.1–5.1, n = 3 studies [19,20,22]). Comorbidities, such as cardiovascular and metabolic conditions, increased BSI risk (OR: 2.1, 95% CI: 1.5–2.9, I^2^ = 52%, prediction interval: 1.2–3.7, n = 7 studies [6,7,11,19,20,25,28]), as did advanced age (>65 years, OR: 1.9, 95% CI: 1.5–2.4, I^2^ = 47%, prediction interval: 1.1–3.3, n = 7 studies [7,11,18,19,20,27,28]). These associations, derived from observational data, do not imply causation due to potential confounders, such as disease severity or healthcare setting. Higher heterogeneity for immunosuppression (I^2^ = 57%) may stem from varying definitions (e.g., corticosteroids vs. other therapies), while limited data for corticosteroid use (n = 2 studies) restricted heterogeneity assessment. Results are summarized in Figure 5.

### 3.5. Clinical Outcomes

**Figure 6:**
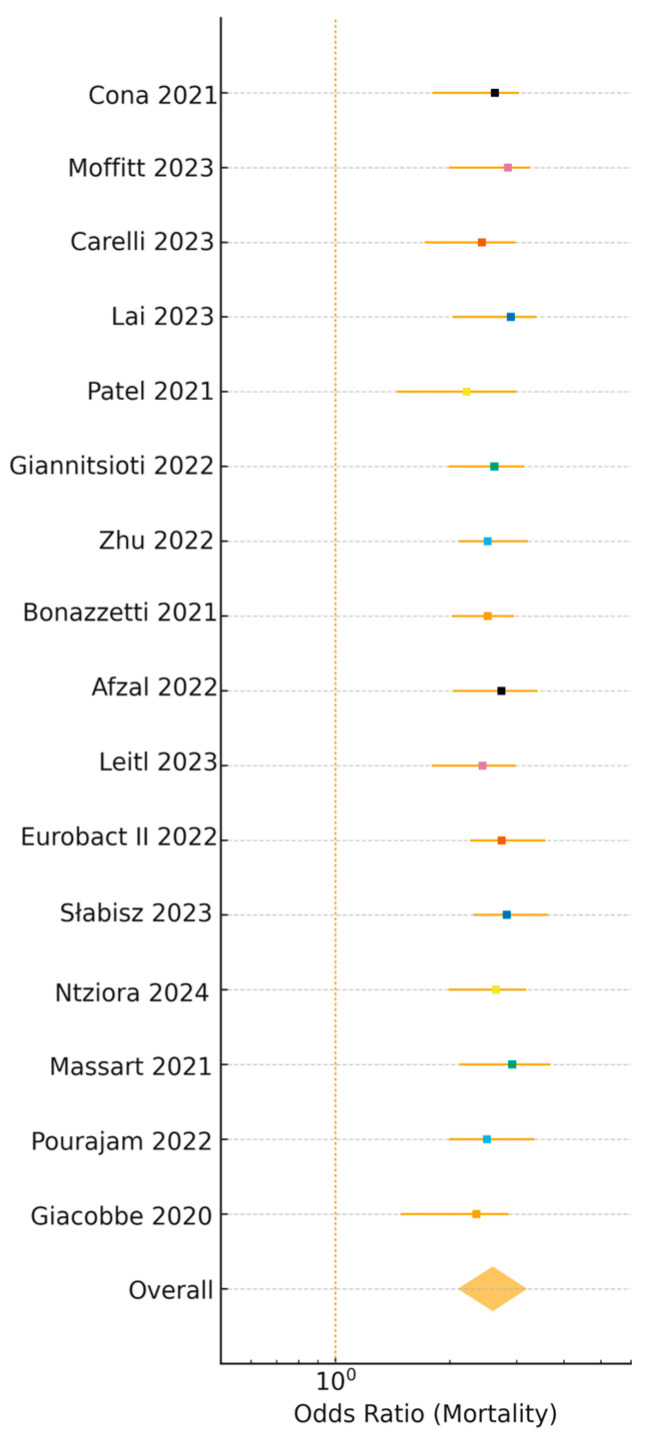
Forest plot of odds ratios (ORs) for mortality associated with bloodstream infections (BSIs) in COVID-19 patients across 16 studies (n = ~123,500 patients). The pooled OR is 2.6 (95% CI: 2.1–3.2, I^2^ = 47%), with the diamond representing the pooled estimate. Point estimates and 95% confidence intervals (CI) are shown, indicating a consistently elevated mortality risk in BSI patients. Data from references [3,4,6,7,8,10,11,13,16,18,19,21,23,24,26,27,28].

BSIs were associated with a 2.6-fold higher mortality risk (OR: 2.6, 95% CI: 2.1–3.2, I^2^ = 47%, prediction interval: 1.6–4.1, n = 16 studies [3,4,6,8,10,11,13,16,18,19,21,23,24,26,27,28]), prolonged hospital stays (mean difference: 6.8 days, 95% CI: 4.8–8.8, I^2^ = 47%, n = 6 studies [6,7,11,20,22,28]), and a 3.1-fold increased likelihood of ICU admission (OR: 3.1, 95% CI: 2.4–4.0, I^2^ = 52%, n = 6 studies [6,7,11,20,22,28]) (see Figure 6, Table 1). These observational associations do not imply causation, as confounders like disease severity may contribute. Pooled mortality among BSI patients was 42.0% (95% CI: 36.0–48.0, I^2^ = 52%, prediction interval: 21.0–66.0%) [3,4,6,8,10,11,13,16,18,19,21,23,24,26,27,28], compared to ~10–20% in non-BSI COVID-19 patients. ICU mortality was highest with multidrug-resistant (MDR) Gram-negative BSIs (>60%) [8,10,27]. Funnel plot analysis showed no significant publication bias (Egger’s test, *p* = 0.16, Supplemental Figure S1).

### 3.6. Study Quality

A total of 22 studies were included in the risk of bias assessment. Of these, 10 were prospective: Giacobbe et al., 2020 [3]; Massart et al., 2021 [6]; Eurobact II, 2022 [11]; Leitl et al., 2023 [13]; Słabisz et al., 2023 [10]; Ntziora & Giannitsioti, 2024 [8]; Cona et al., 2021 [28];Carelli et al., 2023 [14]; Moffitt et al., 2023 [27]; Driedger et al., 2023 [19].

The remaining12 were retrospective: Pourajam et al., 2022 [4]; Shukla et al., 2021 [7]; Bonazzetti et al., 2021 [17]; Giannitsioti et al., 2022 [21]; Afzal et al., 2022 [16]; Patel et al., 2021 [23]; Zhu et al., 2022 [18]; Papić et al., 2024 [24]; Lai et al., 2023 [26]; Fallah et al., 2024 [22]; Zanella et al., 2024 [25]; Montrucchio et al., 2025 [15].

All studies were assessed using the Newcastle–Ottawa Scale (NOS) (domains: selection, comparability, outcome/exposure). Most (20/22) were rated high quality (NOS 7–9), while two studies—Afzal et al., 2022 [16] and Zanella et al., 2024 [25]—were of moderate quality (NOS 5–6) (per-study scores in Supplementary Table S1). Inter-rater agreement was high (Cohen’s Kappa = 0.85). Sensitivity analyses excluding the two moderate-quality studies produced minimal changes in pooled estimates, supporting robustness of the findings (see Table S3).

## 4. Discussion

This meta-analysis of 22 studies (~123,500 patients) provides one of the most comprehensive estimates to date of the burden of BSIs complicating COVID-19. The pooled prevalence of 8.2% is consistent with prior reviews [29,30] and remained stable across early and late pandemic phases. Higher prevalence in ICUs (12.5%) compared with non-ICU settings (5.2%) reflects the role of invasive procedures and critical illness, while pediatric cohorts showed 10.8%, although evidence is limited to three studies.

### 4.1. Comparison with Previous Reviews

Our meta-analysis of 22 studies (n = ~123,500 patients) reports a pooled BSI prevalence of 8.2% (95% CI: 5.7–11.0, I^2^ = 50%), consistent with prior reviews (6–8% [29]), but includes recent prospective data (2023–2025) and pediatric cohorts (10.8%, 95% CI: 6.5–15.5, n = 3 studies [20,26,27]) (see Table 1). Unlike Ippolito et al. (2021) [29], which lacked detailed AMR profiling, we report elevated MRSA (36%, 95% CI: 29–43%) and ESBL-producing Enterobacterales (31%, 95% CI: 25–37%) rates, driven by increased empirical antibiotic use and disrupted infection control during pandemic surges [31,32]. These exceed pre-pandemic European estimates (ECDC, 2023) and align with WHO GLASS data for low- and middle-income countries [31]. In ICUs, MDR Gram-negative organisms reached 40–45% in some cohorts [6,9,15], highlighting AMR challenges. Recent studies (e.g., Sleziak et al., 2025 [9]; Montrucchio et al., 2025 [15]) provide post-Omicron insights, supporting antimicrobial stewardship needs.

### 4.2. Context of Antimicrobial Resistance

The elevated MRSA (36% vs. 25% pre-pandemic [33]) and ESBL (31%) rates likely reflect increased empirical antibiotic use and disrupted infection control during pandemic surges, consistent with WHO GLASS data for low- and middle-income countries (LMICs) [31]. These rates exceed pre-pandemic European estimates (ECDC, 2023 [33]). In ICUs, multidrug-resistant (MDR) Gram-negative organisms reached 40–45% in some cohorts [6,9,14], amplifying AMR challenges. However, retrospective designs in 12/22 studies may overestimate AMR due to selective sampling in sicker patients, while the predominance of high-income country studies (e.g., USA, Italy [3,7,16,17]) may underestimate AMR compared to LMICs, where resistance is typically higher due to variable infection control practices [31,34]. The predominance of Gram-negative pathogens (*Klebsiella pneumoniae* 26%, *Acinetobacter baumannii* 21%) aligns with pre-COVID-19 ICU BSI trends but underscores the pandemic’s exacerbation of resistance [33]. Geographical variations, particularly for *Acinetobacter baumannii*, showed higher resistance in Asia-Pacific and Latin America [4,20,22] compared to North America and Europe [3,7,16,17], reflecting regional prescribing patterns. For *Pseudomonas aeruginosa*, regional differences in resistance across Chinese provinces suggest local practices influence patterns [35]. Fungal BSIs, such as *Candida* spp., may be underreported (grouped under ‘other pathogens,’ 22%), with recent evidence suggesting increased candidemia in severe COVID-19 patients, warranting further investigation [35].

### 4.3. Heterogeneity in BSI Prevalence

Moderate heterogeneity (I^2^ = 50%) in BSI prevalence likely stems from differences in diagnostic criteria, with CDC’s stricter requirement of two positive blood cultures for contaminants potentially underestimating prevalence compared to ECDC’s broader clinical and laboratory criteria (meta-regression, *p* = 0.05). This variability particularly affects classification of coagulase-negative staphylococci (CoNS), where clinical correlation is essential. Subgroup analyses by geographic region showed slight variations: Europe (7.8%), North America (8.5%), Asia-Pacific (9.0%), and Latin America (10.2%) (see Section 3.2), possibly due to regional AMR and infection control differences [34]. Prevalence was stable across early (2020–2021, 8.0%) and later (2022–2025, 8.5%) pandemic phases, suggesting persistent infection prevention challenges. Sensitivity analyses excluding non-standard definitions confirmed robust estimates (see Table S3). Future studies should adopt ECDC criteria for greater sensitivity and report CoNS validation to enhance comparability.

### 4.4. Risk Factors and Clinical Implications

Factors associated with BSIs include mechanical ventilation (OR: 2.6), immunosuppression (OR: 2.3), and corticosteroid use (OR: 2.4), suggesting invasive procedures and immune compromise increase risk [3,6,17,20,23,24]. Pediatric MIS-C was linked to higher BSI risk (OR: 2.4, n = 3 studies [19,20,22]). These observational associations do not confirm causation due to potential confounders like disease severity. Clinicians should prioritize infection prevention, early BSI detection, and targeted antimicrobials (e.g., vancomycin for MRSA, carbapenems for ESBL) based on local resistance profiles.

### 4.5. Strengths and Limitations

This meta-analysis has several strengths, including a large sample size (~123,500 patients, ~602,000 blood cultures) across 22 studies from 12 countries [3,4,6,7,8,10,11,13,14,15,16,17,18,19,20,21,22,23,24,25,26,27,28], inclusion of prospective and retrospective designs, and adherence to PRISMA 2020 guidelines [12]. Moderate heterogeneity (I^2^ = 50%) reflects diverse populations and settings, enhancing generalizability. However, limitations include moderate heterogeneity due to differences in CDC and ECDC diagnostic criteria (meta-regression, *p* = 0.05), which may affect prevalence estimates. Retrospective designs in 12/22 studies may introduce selection bias, particularly from selective blood culture sampling in critically ill patients. Pediatric data are limited to three studies [20,26,27], precluding firm conclusions about MIS-C and BSI risk. Most studies originated from high-income countries (e.g., USA, Italy [3,7,16,17]), potentially underestimating AMR in LMICs, where resistance is typically higher due to resource constraints and variable infection control practices [31]. For instance, WHO GLASS data report elevated ESBL rates in LMICs [31], suggesting our AMR estimates (e.g., 31% ESBL) may not fully capture the burden in these settings. The absence of a formal GRADE assessmentlimits confidence in pooled estimates. Future studies should incorporate GRADE, prioritize LMIC representation, and use harmonized ECDC criteria to enhance comparability.

### 4.6. Implications for Practice and Research

The convergence of COVID-19 and AMR highlights the urgency of strengthening infection prevention and control (IPC) programs, particularly in ICUs. Rapid diagnostics (e.g., next-generation sequencing) and antimicrobial stewardship, aligned with WHO’s AMR action plan [32,36], are critical to mitigate BSIs. Global health policies should integrate AMR surveillance with standardized BSI definitions to enhance data comparability. Prospective, multicenter studies with harmonized criteria and a focus on pediatric populations are essential to address data gaps and guide future pandemics.

## 5. Conclusions

This systematic review and meta-analysis of 22 studies (~123,500 patients, ~602,000 blood cultures) confirms that bloodstream infections (BSIs) are a frequent and severe complication of COVID-19. The pooled prevalence was 8.2%, with higher rates in ICU settings (12.5%) compared to non-ICU populations (5.2%). Pediatric cohorts (Fallah et al., 2024 [22]; Lai et al., 2023 [26]; Moffitt et al., 2023 [27]) showed a prevalence of 10.8%, though limited evidence precludes firm conclusions. While MIS-C appeared associated with an increased risk of BSI, evidence is limited to three studies. Therefore, pediatric-specific findings should be interpreted with caution until validated by larger, multicenter cohorts.

BSIs were associated with a 2.6-fold increased risk of mortality, prolonged hospital stays (+6.8 days), and higher ICU admissions (OR: 3.1). Multidrug-resistant organisms—including MRSA (36%), ESBL-producing *Enterobacterales* (31%), and carbapenem-resistant Gram-negative pathogens—dominated the microbiological profile.

Limitations of the present review include moderate heterogeneity (CDC vs. ECDC diagnostic criteria), the predominance of retrospective designs (12/22 studies), and limited pediatric evidence. Certainty assessment (GRADE) was not performed, which may limit confidence in pooled estimates.

Strengthened infection prevention, antimicrobial stewardship, and harmonized diagnostic definitions are urgently needed. Future research should prioritize multicenter, prospective studies, particularly in low- and middle-income countries, and dedicated pediatric cohorts to address current knowledge gaps.

## Figures and Tables

**Table 1:** Summary of key outcomes from the meta-analysis of bloodstream infections (BSIs) in COVID-19 patients (n = ~123,500 patients). Outcomes include prevalence (%), odds ratios (OR) for risk factors and mortality, and mean differences (MD) in hospital stay (days). Estimates include 95% confidence intervals (CI), I^2^ for heterogeneity (%), prediction intervals, *p*-values, and contributing studies. Abbreviations: BSI = Bloodstream Infection, OR = Odds Ratio, MD = Mean Difference, CI = Confidence Interval. Data from references [3,4,6,7,8,10,13,14,15,16,17,18,19,20,21,22,23,24,25,26,27,28].

Outcome	Pooled Estimate	95% CI	Prediction Interval	*p*-Value	I^2^ (%)	Studies
Prevalence Outcomes						
BSI Prevalence (Overall)	8.2%	5.7–11.0	3.0–15.5	<0.001	50	22 [3,4,6,7,8,10,13,14,15,16,17,18,19,20,21,22,23,24,25,26,27,28]
BSI Prevalence (ICU)	12.5%	9.0–16.5	5.5–21.0	<0.001	42	12 [3,6,7,8,10,11,13,14,15,17,23,24]
BSI Prevalence (Non-ICU)	5.2%	3.4–7.4	2.2–10.5	<0.001	47	7 [4,16,18,19,21,26,28]
BSI Prevalence (Pediatric)	10.8%	6.5–15.5	4.5–18.5	<0.001	40	3 [19,20,22]
Risk Factor Odds Ratios						
Mortality (OR)	2.6	2.1–3.2	1.6–4.1	<0.001	47	16 [3,4,6,8,10,11,13,16,18,19,21,23,24,26,27,28]
Mechanical Ventilation (OR)	2.6	2.0–3.3	1.6–4.1	<0.001	52	10 [3,6,7,11,17,20,23,26,27,28]
Immunosuppression (OR)	2.3	1.7–3.0	1.4–3.6	<0.001	57	6 [3,6,17,20,23,24]
MIS-C (Pediatric, OR)	2.4	1.6–3.7	1.1–5.1	<0.001	42	3 [19,20,22]
Comorbidities (OR)	2.1	1.5–2.9	1.2–3.7	<0.001	52	7 [6,7,11,19,20,22,28]
Clinical Outcome Measures						
Prolonged Hospitalization (MD, days)	6.8	4.8–8.8	3.2–10.5	<0.001	47	6 [6,7,11,20,22,28]
ICU Admission (OR)	3.1	2.4–4.0	1.8–5.4	<0.001	52	6 [6,7,11,20,22,28]

**Table 2:** Distribution of Bloodstream Infection (BSI) Pathogens in COVID-19 Patients.

Pathogen	Pooled Proportion (%)	95% CI	Number of Isolates	Studies	Notes
*Klebsiella pneumoniae*	26	21–31	1560	18 [3,4,6,7,8,10,11,13,14,15,16,17,18,20,21,23,24,26,27,28]	Predominant Gram-negative pathogen.
*Acinetobacter baumannii*	21	16–26	1260	18 [3,4,6,7,8,10,11,13,14,15,16,17,18,20,21,23,24,26,27,28]	Higher prevalence in ICUs; geographical variation noted.
*Enterococcus* spp.	18	15.5–20.5	1080	18 [3,4,6,7,8,10,11,13,14,15,16,17,18,20,21,23,24,26,27,28]	Common in ECMO cohorts.
*Staphylococcus aureus*	13	10.5–15.5	780	18 [3,4,6,7,8,10,11,13,14,15,16,17,18,20,21,23,24,26,27,28]	MRSA rate: 36% (95% CI: 29–43).
Coagulase-negative staphylococci (CoNS)	18	Not reported	1080	18 [3,4,6,7,8,10,11,13,14,15,16,17,18,20,21,23,24,26,27,28]	Potential contaminants; requires clinical correlation.
*Pseudomonas aeruginosa*	5	3–8	300	10 [3,4,6,8,10,16,20,26,27,28]	Geographical variation noted.
*Escherichia coli*	4	2–7	240	10 [3,4,6,8,10,16,20,26,27,28]	ESBL rate: 31% (95% CI: 25–37).
*Candida* spp.	2	1–4	120	5 [8,10,26,27,28]	Limited data; increased in severe COVID-19.
Other pathogens *	22	Not reported	1320	18 [3,4,6,7,8,10,11,13,14,15,16,17,18,20,21,23,24,26,27,28]	Includes *Enterobacter* spp., *Proteus* spp., and other rare isolates.

* Other pathogensincluded *Enterobacter* spp., *Proteus* spp., and rare isolates not individually categorized. *Candida* spp. was analyzed separately but was also sometimes reported under ‘other pathogens’ in the original studies.

## Data Availability

The reproducible R code (Appendix S2) and full extracted dataset (Table S2) are provided in the Supplementary Materials. To ensure long-term accessibility, these materials will also be deposited in a permanent, citable repository upon acceptance.

## Supplementary Materials

The following supporting information can be downloaded at: https://www.mdpi.com/article/10.3390/v17101353/s1, Figure S1: Funnel plot with trim-and-fill, showing no significant publication bias (Egger’s test, *p* = 0.16); Table S1: Detailed NOS Scores; Table S2: Full Data Extraction Spreadsheet; Table S3: Sensitivity Analysis Results; Table S4: Detailed OR Sources for Risk Factors; Appendix S1: Search Strings; Appendix S2: Full R Code, annotated for reproducibility; Appendix S3: Meta-Regression Outputs. The PRISMA 2020 checklist is available upon request from the corresponding author to confirm adherence to reporting guidelines.

## Abbreviations

The following abbreviations are used in this manuscript:

Abbreviation	Full Term
AMR	Antimicrobial Resistance
BSI	Bloodstream Infection
CDC	Centers for Disease Control and Prevention
CI	Confidence Interval
CLABSI	Central Line-Associated Bloodstream Infection
CoNS	Coagulase-Negative Staphylococci
COVID-19	Coronavirus Disease 2019
ESBL	Extended-Spectrum Beta-Lactamase
ICU	Intensive Care Unit
MDRO	Multidrug-Resistant Organism
MIS-C	Multisystem Inflammatory Syndrome in Children
MRSA	Methicillin-Resistant *Staphylococcus aureus*
NOS	Newcastle–Ottawa Scale
OR	Odds Ratio
PRISMA	Preferred Reporting Items for Systematic Reviews and Meta-Analyses
RT-PCR	Reverse Transcription Polymerase Chain Reaction
SARS-CoV-2	Severe Acute Respiratory Syndrome Coronavirus 2
WHO	World Health Organization

## References

[1] Chu H., Chan J.F.-W., Yuen T.T.-T., Shuai H., Yuan S., Wang Y., Hu B., Yip C.C., Tsang J.O., Huang X. (2020). Comparative tropism, replication kinetics, and cell damage profiling of SARS-CoV-2 and SARS-CoV with implications for clinical manifestations, transmissibility, and laboratory studies of COVID-19: An observational study. Lancet Microbe.

[2] Wu Z., McGoogan J.M. (2020). Characteristics of and Important Lessons from the Coronavirus Disease 2019 (COVID-19) Outbreak in China: Summary of a Report of 72,314 Cases from the Chinese Center for Disease Control and Prevention. JAMA.

[3] Giacobbe D.R., Battaglini D., Ball L., Brunetti I., Bruzzone B., Codda G., Crea F., De Maria A., Dentone C., Di Biagio A. (2020). Bloodstream Infections in Critically Ill Patients with COVID-19. Eur. J. Clin. Investig..

[4] Pourajam S., Kalantari E., Talebzadeh H., Mellali H., Sami R., Soltaninejad F., Amra B., Sajadi M., Alenaseri M., Kalantari F. (2022). Secondary Bacterial Infection in COVID-19 Patients. Front. Cell. Infect. Microbiol..

[5] Russell C.D., Fairfield C.J., Drake T.M., Turtle L., Seaton R.A., Wootton D.G., Sigfrid L., Harrison E.M., Docherty A.B., de Silva T.I. (2021). Co-infections, secondary infections, and antimicrobial use in patients hospitalised with COVID-19 during the first pandemic wave from the ISARIC WHO CCP-UK study: A multicentre, prospective cohort study. Lancet Microbe.

[6] Massart N., Maxime V., Fillatre P., Razazi K., Ferré A., Moine P., Legay F., Voiriot G., Amara M., Santi F. (2021). Characteristics and Prognosis of Bloodstream Infection in Patients with COVID-19 Admitted in the ICU: An Ancillary Study of the COVIDICU Study. Ann. Intensive Care.

[7] Shukla B.S., Warde P.R., Knott E., Arenas S., Pronty D., Ramirez R., Rego A., Jimenez G.S., Larson E.L., Pereira M.R. (2021). Bloodstream Infection Risk, Incidence, and Deaths for Adults Hospitalized with COVID-19. Emerg. Infect. Dis..

[8] Ntziora F., Giannitsioti E. (2024). Bloodstream infections in the era of the COVID-19 pandemic: Changing epidemiology of antimicrobial resistance in the intensive care unit. J. Intensive Med..

[9] Sleziak J., Błażejewska M., Duszyńska W. (2025). Central Line-Associated Bloodstream Infections in Intensive Care Unit During and After the COVID-19 Pandemic, 5-Year Prospective Observational Study. J. Clin. Med..

[10] Słabisz N., Dudek-Wicher R., Leśnik P., Majda J., Kujawa K., Nawrot U. (2023). Impact of the COVID-19 Pandemic on the Epidemiology of Bloodstream Infections in Hospitalized Patients—Experience from a 4th Military Clinical Hospital in Poland. J. Clin. Med..

[11] Buetti N., Tabah A., Loiodice A., Ruckly S., Aslan A.T., Montrucchio G., Cortegiani A., Saltoglu N., Kayaaslan B., Aksoy F. (2022). Different epidemiology of bloodstream infections in COVID-19 compared to non-COVID-19 critically ill patients: A descriptive analysis of the Eurobact II study. Crit. Care.

[12] Page M.J., McKenzie J.E., Bossuyt P.M., Boutron I., Hoffmann T.C., Mulrow C.D., Shamseer L., Tetzlaff J.M., Akl E.A., Brennan S.E. (2021). The PRISMA 2020 statement: An updated guideline for reporting systematic reviews. BMJ.

[13] Leitl C.J., Stoll S.E., Wetsch W.A., Kammerer T., Mathes A., Böttiger B.W., Seifert H., Dusse F. (2023). Next-Generation Sequencing in Critically Ill COVID-19 Patients with Suspected Bloodstream Infections. J. Clin. Med..

[14] Carelli S., Dell’Anna A.M., Montini L., Bernardi G., Gozza M., Cutuli S.L., Natalini D., Bongiovanni F., Tanzarella E.S., Pintaudi G. (2023). Bloodstream infections in COVID-19 patients undergoing extracorporeal membrane oxygenation in ICU: An observational cohort study. Heart Lung.

[15] Montrucchio G., Grillo F., Balzani E., Gavanna G., Sales G., Bonetto C., Simonetti U., Zanierato M., Fanelli V., Filippini C. (2025). Impact of Multidrug-Resistant Bacteria in a Cohort of COVID-19 Critically Ill Patients: Data from a Prospective Observational Study Conducted in a High-Antimicrobial-Resistance-Prevalence Center. J. Clin. Med..

[16] Afzal A., Perez Gutierrez V., Gomez E., Mon A.M., Moreira Sarmiento C., Khalid A., Polishchuk S., Al-Khateeb M., Yankulova B., Yusuf M. (2022). Bloodstream infections in hospitalized patients before and during the COVID-19 surge in a community hospital in the South Bronx. Int. J. Infect. Dis..

[17] Bonazzetti C., Morena V., Giacomelli A., Oreni L., Casalini G., Galimberti L.R., Bolis M., Rimoldi M., Ballone E., Colombo R. (2021). Unexpectedly high frequency of enterococcal bloodstream infections in coronavirus disease 2019 patients admitted to an Italian ICU: An observational study. Crit. Care Med..

[18] Zhu N.J., Rawson T.M., Mookerjee S., Price J.R., Davies F., Otter J., Aylin P., Hope R., Gilchrist M., Shersing Y. (2022). Changing patterns of bloodstream infections in the community and acute care across two COVID-19 epidemic waves: A retrospective analysis using data linkage. Clin. Infect. Dis..

[19] Driedger M., Daneman N., Brown K., Gold W.L., Jorgensen S.C.J., Maxwell C., Schwartz K.L., Morris A.M., Thiruchelvam D., Langford B. (2023). The impact of the COVID-19 pandemic on blood culture practices and bloodstream infections. Microbiol. Spectr..

[20] Santos C.V., Fukushima E.A., Zhao W., Sharma M., Youssef D., Spzunar S., Levine M., Saravolatz L., Bhargava A. (2022). Incidence of bloodstream infections in patients with COVID-19: A retrospective cohort study of risk factors and outcomes. Germs.

[21] Giannitsioti E., Louka C., Mamali V., Kousouli E., Velentza L., Papadouli V., Loizos G., Mavroudis P., Kranidiotis G., Rekleiti N. (2022). Bloodstream Infections in a COVID-19 Non-ICU Department: Microbial Epidemiology, Resistance Profiles and Comparative Analysis of Risk Factors and Patients’ Outcome. Microorganisms.

[22] Fallah F., Karimi A., Azimi L., Ghandchi G., Gholinejad Z., Abdollahi N., AhariOskooie N., Khodaei H., Armin S., Behzad A. (2024). The impact of the COVID-19 pandemic on pediatric bloodstream infections and alteration in antimicrobial resistance phenotypes in Gram-positive bacteria, 2020–2022. BMC Pediatr..

[23] Patel P.R., Weiner-Lastinger L.M., Dudeck M.A., Fike L.V., Kuhar D.T., Edwards J.R., Pollock D., Benin A. (2022). Impact of COVID-19 pandemic on central-line-associated bloodstream infections during the early months of 2020, National Healthcare Safety Network. Infect. Control Hosp. Epidemiol..

[24] Papic I., Bistrovic P., Cikara T., Busic N., Keres T., Ortner Hadziabdic M., Lucijanic M. (2024). Corticosteroid Dosing Level, Incidence and Profile of Bacterial Blood Stream Infections in Hospitalized COVID-19 Patients. Viruses.

[25] Zanella M.-C., Pianca E., Catho G., Obama B., DeKraker M.E.A., Nguyen A., Chraiti M.-N., Sobel J., Fortchantre L., Harbarth S. (2024). Increased Peripheral Venous Catheter Bloodstream Infections during COVID-19 Pandemic, Switzerland. Emerg. Infect. Dis..

[26] Lai H.C., Hsu Y.L., Lin C.H., Wei H.M., Chen J.A., Low Y.Y., Chiu Y.T., Lin H.C., Hwang K.P. (2023). Bacterial coinfections in hospitalized children with COVID-19 during the SARS-CoV-2 Omicron BA.2 variant pandemic in Taiwan. Front. Med..

[27] Moffitt K.L., Nakamura M.M., Young C.C., Newhams M.M., Halasa N.B., Reed J.N., Fitzgerald J.C., Spinella P.C., Soma V.L., Walker T.C. (2023). Community-Onset Bacterial Coinfection in Children Critically Ill with Severe Acute Respiratory Syndrome Coronavirus 2 Infection. Open Forum Infect. Dis..

[28] Cona A., Tavelli A., Renzelli A., Varisco B., Bai F., Tesoro D., Za A., Biassoni C., Battaglioli L., Allegrini M. (2021). Incidence, Risk Factors and Impact on Clinical Outcomes of Bloodstream Infections in Patients Hospitalised with COVID-19: A Prospective Cohort Study. Antibiotics.

[29] Ippolito M., Simone B., Filisina C., Catalanotto F.R., Catalisano G., Marino C., Misseri G., Giarratano A., Cortegiani A. (2021). Bloodstream Infections in Hospitalized Patients with COVID-19: A Systematic Review and Meta-Analysis. Microorganisms.

[30] Damonti L., Gasser M., Kronenberg A., Buetti N. (2024). Epidemiology of bloodstream infections caused by extended-spectrum cephalosporin-resistant *Escherichia coli* and *Klebsiella pneumoniae*. J. Hosp. Infect..

[31] World Health Organization (2022). Global Antimicrobial Resistance and Use Surveillance System (GLASS) Report 2022.

[32] World Health Organization (2015). Global Action Plan on Antimicrobial Resistance.

[33] European Centre for Disease Prevention and Control (2023). Antimicrobial Resistance in the EU/EEA (EARS-Net)—Annual Epidemiological Report 2023.

[34] Xiao S., Liang X., Han L., Zhao S. (2024). Incidence, antimicrobial resistance and mortality of *Pseudomonas aeruginosa* bloodstream infections among hospitalized patients in China: A retrospective observational multicenter cohort study from 2017 to 2021. Front. Public Health.

[35] Srisurapanont K., Lerttiendamrong B., Meejun T., Thanakitcharu J., Manothummetha K., Thongkam A., Chuleerarux N., Sanguankeo A., Li L.X., Leksuwankun S. (2024). Candidemia Following Severe COVID-19 in Hospitalised and Critical Ill Patients: A Systematic Review and Meta-Analysis. Mycoses.

[36] Lob S.H., Hoban D.J., Sahm D.F., Badal R.E. (2016). Regional differences and trends in antimicrobial susceptibility of *Acinetobacter baumannii*. Int. J. Antimicrob. Agents.

