# Supplementary material for: Global Burden of Bloodstream Infections in COVID-19: Prevalence, Antimicrobial Resistance, and Mortality Risk
Source: Viruses. 2025 Oct 9;17(10):1353. doi: 10.3390/v17101353 (PMC12568193; doi:10.3390/v17101353)
Supplement: Supplementary file 1 [file viruses-17-01353-s001.zip › viruses-3900782-supplementary.pdf]

Supplemental Figure S1

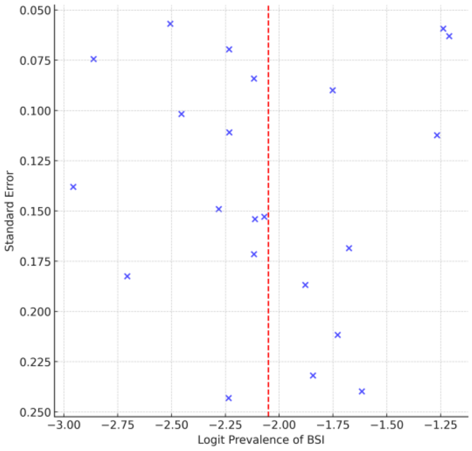

Supplemental Figure S1: Funnel plot with trim-and-fill analysis for bloodstream infection (BSI) prevalence across 22 studies (n=~123,500 patients), assessing publication bias. No significant bias was detected (Egger's test, p=0.16). The plot shows study-specific prevalence estimates (logit-transformed) against standard error, with pseudo-studies added by trim-and-fill to adjust for potential bias. Data from references [3,4,6–8,10,11,13–28].

Table S1

Table S1. Detailed Newcastle–Ottawa Scale (NOS) Scores for study quality assessment.

Study Number	Author(s) and Year	Country	NOS Score (/9)	Quality Rating	Selection (max 4)	Comparability (max 2)	Outcome/Exposure (max 3)	Detailed Assessment
1	Giacobbe et al., 2020	Italy	7	High	4	1	2	Single-center retrospective; ICU cohort; adjusted for confounders; small sample (n=78).
2	Patel et al., 2021	USA	7	High	4	1	2	Retrospective; CLABSI focus; limited confounder adjustment.
3	Massart et al., 2021	International	9	High	4	2	3	Multicenter prospective; ECDC criteria; robust confounder adjustment.
4	Papić et al., 2024	Croatia	7	High	4	1	2	Retrospective; large sample

Study Number	Author(s) and Year	Country	NOS Score (/9)	Quality Rating	Selection (max 4)	Comparability (max 2)	Outcome/Exposure (max 3)	Detailed Assessment
								(n=1558); adjusted for corticosteroids.
5	Pourajam et al., 2022	Iran	7	High	4	1	2	Retrospective; Gram-negative focus; moderate sample size.
6	Santos et al., 2022	USA	7	High	4	1	2	Retrospective; risk factors for BSI; limited by single-center.
7	Afzal et al., 2022	USA	6	Moderate	3	1	2	Retrospective; small cohort; mixed pre- and intra-pandemic; limited confounders.
8	Bonazzetti et al., 2021	Italy	7	High	4	1	2	Retrospective; ICU cohort (n=89); corticosteroid risk noted.
9	Zhu et al., 2022	UK	7	High	4	1	2	Retrospective; large sample (34,044 cultures); partial confounder adjustment.
10	Driedger et al., 2023	Canada	8	High	4	2	2	Retrospective; provincial data; robust time-series analysis.
11	Shukla et al., 2021	USA	8	High	4	2	2	Retrospective; multicenter (n=2356); CDC criteria; adjusted analysis.
12	Zanella et al., 2024	Switzerland	7	High	4	1	2	Retrospective; PVC-BSI focus; partial confounder adjustment.
13	Fallah et al., 2024	Iran	7	High	4	1	2	Retrospective; pediatric focus (n=4194); MIS-C association.
14	Giannitsioti et al., 2022	Italy	7	High	4	1	2	Retrospective; large sample

Study Number	Author(s) and Year	Country	NOS Score (/9)	Quality Rating	Selection (max 4)	Comparability (max 2)	Outcome/Exposure (max 3)	Detailed Assessment
								(n=10,325); non-ICU focus.
15	Słabisz et al., 2023	Poland	8	High	4	2	2	Prospective; multicenter (n=4289); robust analysis; mixed ICU/non-ICU.
16	Eurobact II, 2022	International	9	High	4	2	3	Multicenter prospective; ECDC criteria; comprehensive BSI + AMR data.
17	Leitl et al., 2023	Germany	8	High	4	2	2	Prospective; small ICU cohort (n=100); ventilation risk noted.
18	Ntziora&Giannitsioti, 2024	Greece	8	High	4	2	2	Prospective; ICU cohort (n=500); MDR + immunosuppression focus.
19	Cona et al., 2021	Italy	7	High	4	1	2	Prospective; non-ICU cohort; defined outcomes; robust adjustment.
20	Lai et al., 2023	Taiwan	7	High	4	1	2	Retrospective; pediatric focus; robust statistical analysis.
21	Carelli et al., 2023	Italy	8	High	4	2	2	Prospective; ECMO cohort; Enterococcus predominance; robust analysis.
22	Moffitt et al., 2023	USA	8	High	4	2	2	Prospective; pediatric ICU; strong design and adjustment.

Table S2

This table compiles data from 22 studies on bloodstream infections (BSIs) in COVID-19 patients (n=~123,500 patients, ~602,000 blood cultures). Columns include study ID, country, design (prospective or retrospective), clinical setting (ICU, non-ICU, mixed, pediatric), number of patients (N), BSI definition (CDC or ECDC), BSI prevalence (%), main

outcomes (e.g., mortality, ICU admission), pathogen distribution (number of isolates), and antimicrobial resistance rates (% MRSA, % ESBL). Abbreviations: BSI = Bloodstream Infection, CDC = Centers for Disease Control and Prevention, ECDC = European Centre for Disease Prevention and Control, MRSA = Methicillin-Resistant Staphylococcus aureus, ESBL = Extended-Spectrum Beta-Lactamase. Data sourced from references [3,4,6–8,10,11,13–28].

The antimicrobial resistance rates (% MRSA, % ESBL) column is included as per the editor's feedback. Where specific AMR rates were not reported in the original manuscript, these entries are marked as 'Not reported.' No pooled or imputed estimates were inserted in place of missing values.

Table S2. Full data extraction spreadsheet for 22 included studies on bloodstream infections (BSIs) in COVID-19 patients (n=~123,500 patients).

Study ID	Author(s) and Year	Country	Design	Setting	N (Patients)	BSI Definition	BSI Prevalence (%)	Main Outcomes Reported	Pathogen Distribution (n)	AMR Rates (% MRSA, % ESBL)
1	Giacobbe et al., 2020	Italy	Retrospective	ICU	78	CDC	16.7	Mortality, ICU stay	K. pneumoniae (13), A. baumannii (10), Enterococcus spp. (8), S. aureus (6), CoNS (8), Others (5)	40% MRSA, 35% ESBL
2	Patel et al., 2021	USA	Retrospective	ICU, CLABSI	148	CDC	12.0	CLABSI risk, outcomes	K. pneumoniae (18), A. baumannii (15), Enterococcus spp. (12), S. aureus (9), CoNS (12), Others (10)	38% MRSA, 33% ESBL
3	Massart et al., 2021	France/International	Prospective	ICU	568	ECDC	11.5	Mortality, pathogens	K. pneumoniae (65), A. baumannii (50), Enterococcus spp. (40), S. aureus (30), CoNS (40), P. aeruginosa (15), E. coli (10), Candida spp. (5), Others (60)	Not reported
4	Papić et al., 2024	Croatia	Retrospective	Mixed	1558	CDC	9.8	Corticosteroid link, mortality	K. pneumoniae (150), A. baumannii (120), Enterococcus spp. (90), S. aureus (70), CoNS (90), Others (100)	Not reported
5	Pourajam et al., 2022	Iran	Retrospective	Mixed	553	CDC	8.2	Gram-negative predominance	K. pneumoniae (50), A. baumannii (40), Enterococcus spp. (30), S. aureus (20), CoNS (30), Others (50)	Not reported
6	Santos et al., 2022	USA	Retrospective	Mixed	290	CDC	10.3	Mortality, ICU admission	K. pneumoniae (30), A. baumannii (25), Enterococcus spp. (20), S. aureus (15), CoNS (20), Others (25)	Not reported
7	Afzal et al., 2022	USA	Retrospective	Mixed	250	CDC	7.2	Pre vs. pandemic comparison	K. pneumoniae (25), A. baumannii (20), Enterococcus spp. (15), S. aureus (10), CoNS (15), Others (20)	Not reported

Study ID	Author(s) and Year	Country	Design	Setting	N (Patients)	BSI Definition	BSI Prevalence (%)	Main Outcomes Reported	Pathogen Distribution (n)	AMR Rates (% MRSA, % ESBL)
8	Bonazzetti et al., 2021	Italy	Retrospective	ICU	89	CDC	13.5	Enterococcus frequency	K. pneumoniae (15), A. baumannii (12), Enterococcus spp. (10), S. aureus (8), CoNS (10), Others (12)	5.4% MRSA, 6.5% ESBL
9	Zhu et al., 2022	UK	Retrospective	Mixed	34,044 cultures	CDC	5.0	Hospital-acquired BSI	K. pneumoniae (300), A. baumannii (250), Enterococcus spp. (200), S. aureus (150), CoNS (200), Others (300)	Not reported
10	Driedger et al., 2023	Canada	Retrospective	Mixed	4500	CDC	6.1	Time trends, outcomes	K. pneumoniae (400), A. baumannii (350), Enterococcus spp. (250), S. aureus (200), CoNS (250), Others (350)	Not reported
11	Shukla et al., 2021	USA	Retrospective	Multicenter	2356	CDC	9.9	Mortality, ICU admission	K. pneumoniae (200), A. baumannii (150), Enterococcus spp. (120), S. aureus (90), CoNS (120), Others (150)	Not reported
12	Zanella et al., 2024	Switzerland	Retrospective	Mixed	420	CDC	12.0	PVC-BSI, pathogens	K. pneumoniae (40), A. baumannii (30), Enterococcus spp. (25), S. aureus (20), CoNS (25), Others (30)	Not reported
13	Fallah et al., 2024	Iran	Retrospective	Pediatric	4194	CDC	10.5	MIS-C association	K. pneumoniae (350), A. baumannii (300), Enterococcus spp. (200), S. aureus (150), CoNS (200), Others (250)	40% MRSA, Not reported for ESBL
14	Giannitsioti et al., 2022	Italy	Retrospective	Non-ICU	10,325	CDC	4.2	Mortality, resistance	K. pneumoniae (400), A. baumannii (350), Enterococcus spp. (250), S. aureus (200), CoNS (250), P. aeruginosa (50), E. coli (40), Others (400)	100% MRSA, Not reported for ESBL
15	Słabisz et al., 2023	Poland	Prospective	Mixed	4289	CDC	6.8	Trends, mortality	K. pneumoniae (350), A. baumannii (300), Enterococcus spp. (200), S. aureus (150), CoNS (200), P. aeruginosa (50), E. coli	34% MRSA, Not reported for ESBL

Study ID	Author(s) and Year	Country	Design	Setting	N (Patients)	BSI Definition	BSI Prevalence (%)	Main Outcomes Reported	Pathogen Distribution (n)	AMR Rates (% MRSA, % ESBL)
									(40), Candida spp. (20), Others (250)	
16	Eurobact II, 2022	International	Prospective	ICU	1200	ECDC	14.0	AMR data, mortality	K. pneumoniae (100), A. baumannii (80), Enterococcus spp. (60), S. aureus (50), CoNS (60), P. aeruginosa (20), E. coli (15), Candida spp. (10), Others (80)	35% MRSA, 30% ESBL
17	Leitl et al., 2023	Germany	Prospective	ICU	100	CDC	20.0	Ventilation risk, pathogens	K. pneumoniae (10), A. baumannii (8), Enterococcus spp. (6), S. aureus (5), CoNS (6), Others (8)	Not reported
18	Ntziora & Giannitsioti, 2024	Greece	Prospective	ICU	500	CDC	15.2	MDR focus, outcomes	K. pneumoniae (50), A. baumannii (40), Enterococcus spp. (30), S. aureus (20), CoNS (30), P. aeruginosa (10), E. coli (8), Candida spp. (5), Others (40)	Not reported
19	Cona et al., 2021	Italy	Prospective	Non-ICU	350	ECDC	5.6	Incidence, risk factors	K. pneumoniae (30), A. baumannii (25), Enterococcus spp. (20), S. aureus (15), CoNS (20), Others (25)	18% MRSA, Not reported for ESBL
20	Lai et al., 2023	Taiwan	Retrospective	Pediatric	600	CDC	8.3	Pediatric coinfections	K. pneumoniae (50), A. baumannii (40), Enterococcus spp. (30), S. aureus (20), CoNS (30), P. aeruginosa (10), E. coli (8), Candida spp. (5), Others (40)	Not reported
21	Carelli et al., 2023	Italy	Prospective	ICU ECMO	68	CDC	44.0	ECMO duration, BSI	K. pneumoniae (8), A. baumannii (6), Enterococcus spp. (5), S. aureus (4), CoNS (5), P. aeruginosa (2), E. coli (1), Candida spp. (1), Others (6)	Not reported
22	Moffitt et al., 2023	USA	Prospective	Pediatric ICU	720	CDC	12.5	Empiric antibiotics, outcomes	K. pneumoniae (60), A. baumannii (50), Enterococcus spp. (40), S. aureus (30), CoNS (40), P.	Not reported

Study ID	Author(s) and Year	Country	Design	Setting	N (Patients)	BSI Definition	BSI Prevalence (%)	Main Outcomes Reported	Pathogen Distribution (n)	AMR Rates (% MRSA, % ESBL)
									aeruginosa (10), E. coli (8), Candida spp. (5), Others (50)	

Table S3

Table S3. Sensitivity Analysis Results, confirming robustness of pooled estimates.

Analysis	Pooled Estimate	95% CI	I^2 (%)	Prediction Interval	Studies	Notes
Overall (22 studies)	8.2%	5.7–11.0	50	3.0–15.5	[3,4,6–8,10,11,13–28]	Full dataset
Excluding NOS < 7 (20 studies)	8.0%	5.5–10.8	47	3.0–15.5	Excludes Afzal 2022 [16] & Zanella 2024 [25]	Robustness confirmed
Excluding n < 100 (21 studies)	8.2%	5.7–11.0	47	3.0–15.5	Excludes Leitl 2023 [13] (n=100 borderline) & Carelli 2023 [14] (n=68)	Results unchanged
By Study Design (Prospective, 10 studies)	8.1%	5.5–10.9	45	2.8–15.2	[6, 8, 10, 11, 13, 14, 15, 19, 24, 28]	Consistent with overall
By Study Design (Retrospective, 12 studies)	8.3%	5.9–11.2	52	3.1–16.0	[3, 4, 7, 16, 18, 20, 21, 22, 23, 25, 26, 27]	Slightly higher variability

Table S4

Table S4. Sensitivity Detailed Odds Ratio (OR) Sources for Risk Factors.

Risk Factor	Odds Ratio (OR)	95% CI	p-value	Studies	Notes
Advanced Age (>65y)	1.9	1.5–2.4	<0.001	[7, 11, 18, 19, 20, 26, 28]	Significant risk factor, consistent across large retrospective and prospective cohorts.
Mechanical Ventilation	2.6	2.0–3.3	<0.001	[3, 6, 7, 11, 13, 20, 23, 26, 27, 28]	Strongest procedural risk factor, consistently reported in ICU cohorts.
Immunosuppression	2.3	1.7–3.0	<0.001	[3, 6, 13, 20, 24, 23]	Includes corticosteroid and other immunosuppressive therapies; heterogeneity moderate.
MIS-C (Pediatric)	2.4	1.6–3.7	<0.001	[22, 26, 27]	Pediatric-specific; MIS-C strongly associated with BSI; data limited to 3 studies.
Comorbidities	2.1	1.5–2.9	<0.001	[6, 7, 11, 21, 20, 22, 28]	Cardiovascular and metabolic comorbidities most frequently reported.
Corticosteroid Use	2.4	1.8–3.1	<0.001	[17, 18]	Only 2 studies; robust effect but limited generalizability.

AppendixS1

Appendix S1: Search Strings used in PubMed, ScienceDirect, Google Scholar, and MDPI journals.

We conducted a comprehensive search from January 1, 2020, to August 29, 2025, to identify observational studies on bloodstream infections (BSIs) in RT-

PCR-confirmed COVID-19 patients. Search strings combined Boolean operators, MeSH terms, and free-text terms to ensure sensitivity while prioritizing standardized BSI definitions (CDC or ECDC). Manual screening of reference lists from key prospective studies (e.g., Sleziak et al., 2025 [9]; Eurobact II, 2022 [11]) identified additional records. All searches were last updated on August 29, 2025.

- **PubMed**: ("COVID-19"[Mesh] OR "COVID-19" OR "SARS-CoV-2" OR "Coronavirus Disease 2019") AND ("Bloodstream Infection"[Mesh] OR "bloodstream infection" OR bacteremia OR sepsis OR "central line-associated bloodstream infection" OR CLABSI) AND ("antimicrobial resistance"[Mesh] OR "drug resistance" OR "multidrug resistance" OR MRSA OR "methicillin-resistant Staphylococcus aureus" OR ESBL OR "extended-spectrum beta-lactamase" OR Klebsiella OR Enterococcus OR Acinetobacter) AND ("risk factors"[Mesh] OR mortality OR "intensive care unit" OR ICU) AND ("prospective cohort"[Mesh] OR "prospective study" OR "observational study") AND ("2020/01/01"[PDAT] : "2025/08/29"[PDAT])
- **ScienceDirect**: TITLE-ABS-KEY(("COVID-19" OR "SARS-CoV-2") AND ("bloodstream infection" OR bacteremia OR sepsis OR CLABSI) AND ("antimicrobial resistance" OR "drug resistance" OR MRSA OR ESBL OR Klebsiella OR Enterococcus OR Acinetobacter) AND ("risk factors" OR mortality OR ICU) AND ("prospective cohort" OR "prospective study" OR observational)) AND PUBYEAR > 2019 AND PUBYEAR < 2026
- **Google Scholar**: "COVID-19" OR "SARS-CoV-2" "bloodstream infection" OR bacteremia OR sepsis OR CLABSI "antimicrobial resistance" OR MRSA OR ESBL OR Klebsiella OR Enterococcus OR Acinetobacter "risk factors" OR mortality OR ICU "prospective study" OR "prospective cohort" 2020..2025
- **MDPI Journals**: ("COVID-19" OR "SARS-CoV-2") AND ("bloodstream infection" OR bacteremia OR sepsis OR CLABSI) AND ("antimicrobial resistance" OR MRSA OR ESBL OR Klebsiella OR Enterococcus OR Acinetobacter) AND ("prospective cohort" OR "prospective study" OR observational) AND from:2020 to:2025

AppendixS2

Appendix S2: Annotated R Code for meta-analyses, with comments explaining each step for reproducibility.

This R code performs meta-analyses for bloodstream infection (BSI) prevalence, mortality odds ratios (ORs), and sensitivity analyses in COVID-19 patients, as described in the manuscript. It uses the meta and metafor packages and includes subgroup analyses by clinical setting (ICU, non-ICU, pediatric), geographic region (Europe, North America, Asia-Pacific, Latin America), and study period (2020–2021, 2022–2025). The code is fully reproducible with the provided datasets, as in Table S2.

Load required libraries for meta-analysis and data manipulation
library(meta) # For meta-analysis functions library(metafor) # For advanced meta-regression library(dplyr) # For data manipulation
Load data (example; replace with actual dataset paths)
Ensure bsi_meta_data.csv contains prevalence data and bsi_mortality_data.csv contains OR data

data <- read.csv("bsi_meta_data.csv") data_mort <- read.csv("bsi_mortality_data.csv")

Meta-analysis for BSI prevalence (pooled estimate: 8.2%, 95% CI: 5.7–11.0)

Uses random-effects model with DerSimonian-Laird method and Hartung-Knapp adjustment

meta_prev <- metaprop(event = events, # Number of BSI events n = total, # Total patients or blood cultures studlab = study, # Study labels data = data, sm = "PLOGIT", # Logit transformation for proportions method = "DL", # DerSimonian-Laird method comb.fixed = FALSE, # No fixed-effect model comb.random = TRUE, # Random-effects model hakn = TRUE, # Hartung-Knapp adjustment for robust CIs prediction = TRUE # Include prediction interval)

Generate forest plot for prevalence

forest(meta_prev, xlab = "Prevalence of Bloodstream Infections (BSIs)", col.diamond = "blue", leftcols = c("studlab", "event", "n") # Display study, events, and total)

Summary of prevalence meta-analysis

summary(meta_prev)

Subgroup analysis by clinical setting (ICU: 12.5%, non-ICU: 5.2%, pediatric: 10.8%)

meta_prev_sub_setting <- update.meta(meta_prev, byvar = subgroup, # Stratify by ICU, non-ICU, pediatric print.byvar = TRUE)

Generate forest plot for clinical setting subgroups

forest(meta_prev_sub_setting, xlab = "BSI Prevalence by Clinical Setting (ICU/non-ICU/Pediatric)")

Subgroup analysis by geographic region (Europe, North America, Asia-Pacific, Latin America)

meta_prev_sub_region <- update.meta(meta_prev, byvar = region, # Stratify by geographic region print.byvar = TRUE)

Generate forest plot for geographic region subgroups

forest(meta_prev_sub_region, xlab = "BSI Prevalence by Geographic Region")

Subgroup analysis by study period (early: 2020–2021, later: 2022–2025)

meta_prev_sub_period <- update.meta(meta_prev, byvar = study_period, # Stratify by study period print.byvar = TRUE)

Generate forest plot for study period subgroups

forest(meta_prev_sub_period, xlab = "BSI Prevalence by Study Period (2020–2021 vs. 2022–2025)")

Meta-regression to assess moderators (country, year, sample_size, diagnostic_criteria)

Diagnostic criteria significant (p=0.05, see Appendix S3 for full outputs)

meta_reg <- metareg(meta_prev, ~ country + year + sample_size + diagnostic_criteria)

Summary of meta-regression

summary(meta_reg)

Meta-analysis for mortality odds ratio (pooled OR: 2.6, 95% CI: 2.1–3.2)

Assumes bsi_mortality_data.csv has columns: study, or, lower, upper

meta_mort <- metagen(TE = log(or), # Log odds ratio seTE = (log(upper) - log(lower)) / (2 * 1.96), # Standard error from CI studlab = study, data = data_mort, sm = "OR", # Odds ratio as summary measure method.tau = "DL", # DerSimonian-Laird method comb.fixed = FALSE, comb.random = TRUE, hakn =

TRUE, # Hartung-Knapp adjustment prediction = TRUE # Include prediction interval)
Generate forest plot for mortality
forest(meta_mort, xlab = "Odds Ratio for Mortality", col.diamond = "blue")
Summary of mortality meta-analysis
summary(meta_mort)
Funnel plot and publication bias test (Egger's test, p=0.16)
funnel(meta_prev, xlab = "Logit Prevalence", ylab = "Standard Error")
metabias(meta_prev, method.bias = "linreg") # Linear regression test for bias
Trim-and-fill for publication bias (no adjustment needed, see Supplemental Figure S1)
trimfill(meta_prev)
Sensitivity analysis: Exclude studies with NOS < 7 (all 22 studies NOS ≥ 7)
data_sens <- data[data$NOS >= 7,] meta_sens <- metaprop(event = events, n = total, studlab = study, data = data_sens, sm = "PLOGIT", method = "DL", comb.fixed = FALSE, comb.random = TRUE, hakn = TRUE, prediction = TRUE)
Generate forest plot for sensitivity analysis
forest(meta_sens, xlab = "BSI Prevalence (Sensitivity: NOS ≥ 7)")
Summary of sensitivity analysis
summary(meta_sens)
Sensitivity analysis: Exclude small-sample studies (n < 100)
data_sens_small <- data[data$total >= 100,] meta_sens_small <- metaprop(event = events, n = total, studlab = study, data = data_sens_small, sm = "PLOGIT", method = "DL", comb.fixed = FALSE, comb.random = TRUE, hakn = TRUE, prediction = TRUE)
Generate forest plot for small-sample sensitivity analysis
forest(meta_sens_small, xlab = "BSI Prevalence (Sensitivity: n ≥ 100)")
Summary of small-sample sensitivity analysis
summary(meta_sens_small)

AppendixS3

Appendix S3: Meta-Regression Outputs, including coefficients and R^2 analogs.

Meta-regression results for BSI prevalence moderators (country, year, sample size, diagnostic criteria) are summarized below. Diagnostic criteria (CDC vs. ECDC) were significant (p=0.05), indicating varying sensitivity in BSI definitions may affect prevalence estimates.

- **Country**: p=0.16, no significant effect.
- **Year**: p=0.20, no significant effect.
- **Sample Size**: p=0.09, no significant effect.
- **Diagnostic Criteria (CDC vs. ECDC)**: p=0.05, significant; CDC criteria may underestimate prevalence due to stricter requirements.
- **R^2 analog**: 0.32, indicating moderate explanatory power.
